# Designing an App to Support Measurement-Based Peer Supervision of Frontline Health Workers Delivering Brief Psychosocial Interventions in Texas: Multimethod Study

**DOI:** 10.2196/55205

**Published:** 2024-03-11

**Authors:** Anubhuti Poudyal, Delta-Marie Lewis, Sarah Taha, Alyssa J Martinez, Lauren Magoun, Y Xian Ho, Natali Carmio, John A Naslund, Katherine Sanchez, Neal Lesh, Vikram Patel

**Affiliations:** 1 Department of Sociomedical Sciences Columbia Mailman School of Public Health New York, NY United States; 2 Dimagi, Inc Cambridge, MA United States; 3 Department of Global Health and Social Medicine Harvard Medical School Boston, MA United States; 4 Patient and Community Engaged Research Center Baylor Scott and White Research Institute Dallas, TX United States

**Keywords:** digital technology, mental health, depression, task sharing, nonspecialist providers, peer supervision, therapy quality

## Abstract

**Background:**

The unmet need for mental health care affects millions of Americans. A growing body of evidence in implementation science supports the effectiveness of task sharing in the delivery of brief psychosocial interventions. The digitization of training and processes supporting supervision can rapidly scale up task-shared interventions and enable frontline health workers (FLWs) to learn, master, and deliver interventions with quality and support.

**Objective:**

We aimed to assess the perceived feasibility and acceptability of a novel mobile and web app designed and adapted to support the supervision, training, and quality assurance of FLWs delivering brief psychosocial interventions.

**Methods:**

We followed human-centered design principles to adapt a prototype app for FLWs delivering brief psychosocial interventions for depression, drawing from an app previously designed for use in rural India. Using a multimethod approach, we conducted focus group sessions comprising usability testing and group interviews with FLWs recruited from a large health system in Texas to assess the feasibility and acceptability of the app. The positive System Usability Scale was used to determine the app’s overall usability. We also calculated the participants’ likelihood of recommending the app to others using ratings of 0 to 10 from least to most likely (net promoter score). Focus group transcripts were coded and analyzed thematically, and recommendations were summarized across 4 key domains.

**Results:**

A total of 18 FLWs varying in role and experience with client care participated in the study. Participants found the app to be usable, with an average System Usability Scale score of 72.5 (SD 18.1), consistent with the industry benchmark of 68. Participants’ likelihood of recommending the app ranged from 5 to 10, yielding a net promoter score of 0, indicating medium acceptability. Overall impressions of the app from participants were positive. Most participants (15/18, 83%) found the app easy to access and navigate. The app was considered important to support FLWs in delivering high-quality mental health care services. Participants felt that the app could provide more structure to FLW training and supervision processes through the systematic collection and facilitation of performance-related feedback. Key concerns included privacy-related and time constraints regarding implementing a separate peer supervision mechanism that may add to FLWs’ workloads.

**Conclusions:**

We designed, built, and tested a usable, functional mobile and web app prototype that supports FLW-delivered psychosocial interventions in the United States through a structured supervision mechanism and systematic collection and review of performance measures. The app has the potential to scale the work of FLWs tasked with delivering these interventions to the hardest-to-reach communities they serve. The results of this project will inform future work to evaluate the app’s use and efficacy in real-world settings to support task-shared mental health programs across the United States.

## Introduction

### Background

In the United States, 22.8% of adults have a mental illness, and 5.5% have a serious mental illness [1]. Following the COVID-19 pandemic, the percentage of adults with symptoms of anxiety or depressive disorders has increased substantially, with preliminary data reporting a prevalence of 20% to 32.3% [2,3], although the extent of the increase is still unclear [4]. In 2021, in the United States, only 65.4% of adults with serious mental illness and 47.2% with any mental illness received mental health services [1], and the proportion receiving “minimally adequate” treatment [5], defined as treatment minimally sufficient for common mental disorders, is low [6,7]. In addition to the direct impact on health and well-being, the economic cost of mental illness is high [8]. Worldwide, 418 million disability-adjusted life years and an economic burden of US $5 trillion are associated with mental health disorders [8]. In the United States, the federal government spent US $280 billion on mental health services in 2020 [9]. Despite significant efforts, access to mental health services remains highly limited owing to barriers such as cost and availability of services [9].

The rate of untreated mental health conditions is a major concern. Although psychosocial interventions have shown remarkable effectiveness in supporting individuals with mental conditions [10,11], barriers to accessing services limit the number of people benefiting from such interventions [12]. Access to mental health support varies widely across the country. According to a 2018 study, over 60% of rural counties reported lacking access to a psychiatrist or psychologist [13]. Studies have reported long delays in appointment scheduling [14], with clients sometimes having to wait many months before they are seen; this is especially true for scheduling first-time appointments [15] and has thus become a common issue for mental health care [16,17]. The COVID-19 pandemic has exacerbated the gaps between a need for and access to mental health services [18], with renewed calls to address the shortage of mental health professionals in the United States [19].

To address the gap in providing adequate services for mental health conditions, the World Health Organization and other institutions have advocated for task-sharing approaches, including screening and treatment protocols that frontline health workers (FLWs) can follow to extend the role of mental health specialists wherever possible, particularly in areas where specialist support is nonexistent or inaccessible [20]. Worldwide, there is growing evidence of the effectiveness of psychosocial interventions delivered by FLWs who are not behavioral health specialists [21-24]; frontline interventions have demonstrated effectiveness and cost-effectiveness in addressing mental health in high-income and low-income countries worldwide [25-29].

Task-sharing programs with trained FLWs were initially pioneered and have since been broadly evaluated across various low- and middle-income countries, many with <1 psychiatrist per 100,000 people, rendering specialist access a near impossibility for much of the world’s population [30-33]. Following the concept of “reciprocal innovation,” in which mutual benefit and learnings are derived from innovations demonstrated to be effective in low-income settings and then applied to high-income settings and vice versa [34], task-sharing programs are now being advocated for use in high-income countries experiencing critical shortages of mental health providers and a need for equitable, culturally competent, and value-based care [21,25,35]. Task-sharing programs show great promise for increasing the health system’s capacity to meet the overwhelming demand for mental health care in the United States, particularly in areas with already limited access to specialized care. They can further allow specialists to focus on more complicated cases, potentially reducing delays for those who require more specialized care [36,37]. Scaling up such task-shared programs requires innovative strategies to ensure quality delivery of interventions and support FLWs’ skill enhancement. Using digital tools for training and supervision of FLWs is a promising next step [21] to enhance FLW skills, deliver psychosocial interventions effectively, and provide supportive supervision. Here, we define an FLW as any health worker without specialized training in mental health care who may be tasked with providing mental health support to clients in their communities, which in the United States can include primary care providers, physician assistants, community health workers (CHWs), lay therapists, peer navigators, nurses, midwives, and health coaches [38]. FLW-delivered interventions often have brief core elements (eg, behavioral activation [BA] [21,39]) and are intended to be conducted during FLW visits with clients in the community or primary care settings.

We built an app prototype guided by the principles of measurement-based care and supportive supervision based on an earlier app designed to facilitate supervision for CHWs in rural India [40] to facilitate skill enhancement of FLWs and effective delivery of FLW-led interventions in Texas. Measurement-based care includes methods of monitoring and improving the quality of psychosocial intervention delivery through objective, measurable metrics generated through systematic data collection to monitor client progress and directly inform care decisions [41-43]. Supportive supervision shifts the focus from the supervisor-supervisee dyad to the entire workforce by including self-assessments and assessments by peers, community members, and supervisors [44]. By digitally supporting measurement-based care and supportive supervision, the app can provide scalable access to measurable, evidence-based metrics that can guide assessments by peers as well as oneself and one’s supervisor. The app is designed to support FLWs trained to implement a psychosocial intervention and who can benefit from a metrics-driven, supportive supervision approach to enhance individual performance and ensure intervention fidelity.

### Goal of This Study

In this study, we aimed to assess the feasibility and acceptability of an adapted version of this prototype app in preparation for potential implementation in the United States following the technology acceptance model [45] and learnings from prior work using similar technology in India and other global settings [46].

## Methods

We used human-centered design principles to adapt an app for FLWs in the United States and used a multimethod approach combining usability testing and group interviews to yield collective interpretations of the feasibility and acceptability of the app among potential target end users [47].

### Prototype Design

The design of the app was based on learnings from implementing an existing app in the Promoting Effective Mental Healthcare Through Peer Supervision (PEERS) project built on the CommCare platform and developed as part of a large-scale randomized controlled trial in India to understand whether the quality of mental health care offered by FLWs can be improved through a scalable measurement-based peer supervision model [46,48]. This study builds on evidence that an allied peer supervision model can provide a scalable approach to improve the quality of mental health care available to populations in low-resource settings [46,49]. As of late 2023, over 200 nonspecialist providers or lay counselors in Madhya Pradesh and Goa with appropriate training and supervision in mental health care but no formal mental health qualifications continue to use the app to support the peer supervision component of their work.

In this study, following the PEERS model, we adapted an Android and web app using an iterative prototype design with feedback from an advisory panel of stakeholders and subject matter experts with backgrounds in clinical professional development education, mental health policy, clinical social work, and development and implementation of CHW programs in Texas. Members of the study team held 4 rounds of discussions with 5 advisory panelists to assess face validity and help ensure that potential target users’ input was directly incorporated throughout the design and development process. Through a series of in-depth interviews, advisory panel members initially provided commentary on the original PEERS app, current supervision practices in the United States, and the app’s potential to support the supervision process. Subsequent in-depth interviews probed feedback on the usability of the prototype and considerations for its implementation within the United States. The advisory panel’s feedback included inputs for the design and development of the app and considerations for future implementation.

**Figure 1:**
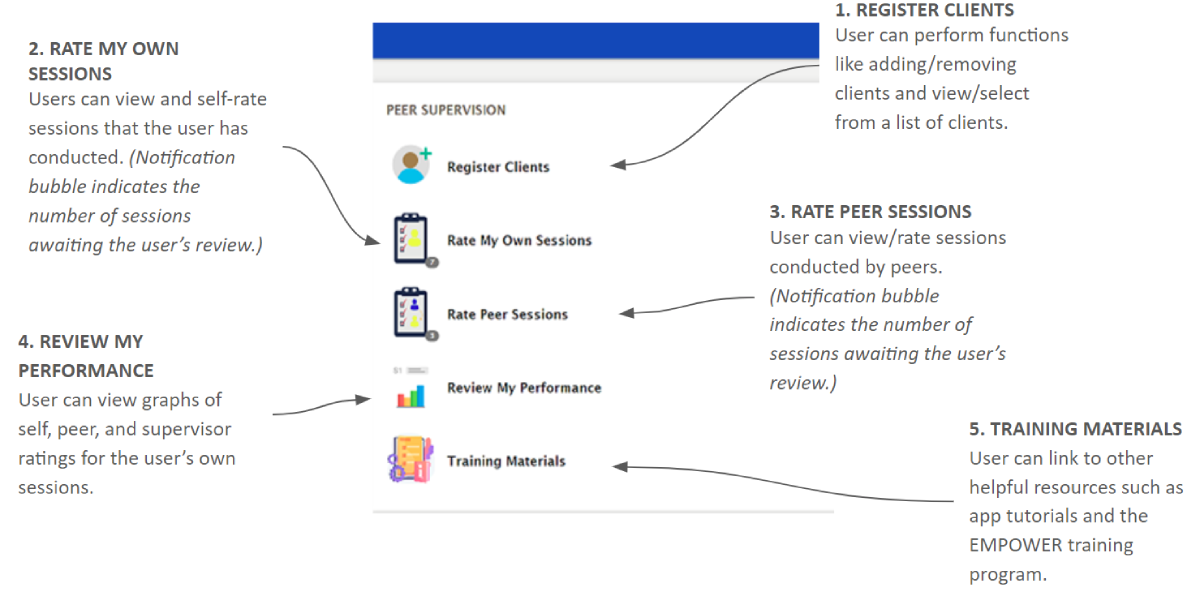
Screenshot of the peer supervision web application menu with descriptions of each menu item.

After incorporating preliminary advisory panel feedback, a final app prototype and study materials were created and prepared for usability testing with target end users. The final app was designed to support the following FLW activities: (1) registering clients, conducting and audio recording intervention sessions, and entering session-related notes; (2) reviewing and rating audio-recorded intervention sessions they conducted themselves using a validated scale (eg, the Behavioral Activation Quality Scale [44]); (3) reviewing and rating audio-recorded intervention sessions conducted by peers; (4) reviewing data visualizations of self-ratings as well as those given by their peers and supervisors for reviewed sessions; and (5) accessing related training materials. A screenshot of the app’s menu and the details of each module are shown in Figure 1.

### Previous Mental Health Training

To assess the feasibility and acceptability of this prototype app, we identified participants who had completed a 2-part training on foundational skills and BA interventions for depression and received access to measurement-based peer supervision training developed by a nonprofit program, EMPOWER, that leverages digital tools for training and supervising FLWs worldwide [46]. The FLWs in this study had been trained as part of efforts to build workforce capacity and scale up access to brief psychosocial interventions for depression in primary care and community settings across Texas [46].

### Recruitment

FLWs from a large health system in Texas (ie, Baylor Scott & White Health) were recruited via email by an experienced human subjects–trained member of the study team to participate in remote focus group sessions. Candidates who met the following eligibility criteria were invited to participate: (1) aged ≥18 years, (2) fluent in English, and (3) having completed training on foundational skills and BA interventions for depression and having access to the measurement-based peer supervision training course developed by EMPOWER. Informed consent was obtained before participation.

### Onboarding to the App

After completing the aforementioned training courses, which included a brief introduction to the app and brief video tutorials on how to use it, participants were invited to join a remote focus group session comprising usability testing and a group interview. All sessions were conducted via Zoom (Zoom Video Communications) and lasted approximately 1.5 hours. Participants who did not own an Android device were mailed a study tablet with the app preinstalled for use during the usability testing portion of the focus group session. Study team members trained in user experience and qualitative methods moderated the sessions (YXH and DML).

### Usability Testing

To assess the app’s usability, a set of 11 usability tasks and related scenarios were created based on tasks we anticipated FLWs would commonly perform if they were to use the app in practice (see the task list in Multimedia Appendix 1). One moderator (DML) introduced each task to the participants, who then performed each task independently at their own pace. The next task was introduced once everyone had completed the preceding one unless the task took much longer than anticipated, in which case the moderator then moved participants to the next task. Moderators observed and noted participants’ task performance while providing minimal assistance with task instructions when needed.

### Surveys

In addition to usability testing, each participant independently completed a series of surveys via Qualtrics (Qualtrics International Inc) to collect information on participant demographics; experience with technology; and perceived ease of use, usefulness, and user satisfaction. We used the positive System Usability Scale (SUS) [50], a 10-item Likert scale survey to assess general usability, with possible scores ranging from 0 to 100, where higher scores indicate higher usability. We also calculated the net promoter score (NPS) [51], a single-item user-reported questionnaire to rate the likelihood of recommending the app to others to assess market viability. To generate an NPS, respondents provide scores between 0 (*not at all likely*) and 10 (*extremely likely*); scores of 9 or 10 are considered “promoters,” scores of 7 or 8 are considered “passives,” and scores of 0 to 6 are considered “detractors.” The NPS is then derived by subtracting detractors from promoters (both calculated as the percentage of total respondents), with possible NPS scores ranging from −100 to 100.

### Group Interviews

All participants engaged in semistructured interviews following usability testing and survey completion. An interview guide was developed and used to collect feedback on user experience with probes on the overall impressions of the app, current supervision mechanisms, and barriers to and facilitators of the broader implementation of the app. A trained and experienced moderator (YXH) followed the guide to probe feedback on the app’s perceived feasibility, acceptability, and usefulness.

### Data Analysis

The focus group sessions were audio recorded, transcribed, and anonymized before coding and analysis. The COREQ (Consolidated Criteria for Reporting Qualitative Research) checklist is included in Multimedia Appendix 2 [52].

We used both deductive and inductive approaches to coding. We first defined usability, feasibility, and acceptability following definitions published by Ginsburg et al [53]. In total, 2 coauthors (YXH and DML) who moderated the focus group sessions independently read 2 transcripts and generated codes from the data. YXH, AP, and DML then finalized the codebook, which was used to direct the coding process. We used a systematic thematic coding approach [54] to generate key themes based on a priori codes and emergent codes: (1) perceived feasibility of the app, (2) acceptability of the app, (3) perceived usefulness of the app, and (4) barriers to and facilitators of scalability. Textbox 1 summarizes the definitions of the themes as used in this paper. Following thematic coding, we developed code summaries or preliminary narratives describing each theme. YXH, AP, and DML then discussed and refined the code summaries to ensure depth and breadth across all participants. The final code summaries were used to describe the findings in this paper. An intercoder agreement was calculated between 2 coders (YXH and DML) on a portion of coded data until a κ value of 0.61 was achieved. All interview transcripts were double coded and analyzed using the qualitative research software Dedoose (version 9.0.107; SocioCultural Research Consultants) [55]. SUS and NPS scores were calculated following the focus group sessions.

**Key themes and definitions.**

**Perceived feasibility**

- Perceived structural factors that could influence the introduction of the app, including administrative infrastructure and operational capabilities of the health systems

**Acceptability**

- Frontline health workers’ willingness to use the app during client interactions

**Perceived usefulness**

- Includes the ability to use the app for enhanced functioning and improvement of the current workflow

**Barriers to and facilitators of scalability**

- Any challenges that could limit scaling up or wider use of the app
- Improvements to the current app, along with changes in the current systems that could result in wider adoption of the app across current and wider health systems

### Ethical Considerations

The institutional review boards at Baylor Scott & White Health (388136) and Harvard Medical School (IRB22-0692) approved the study.

## Results

### Overview

A total of 18 FLWs—including clinical managers, CHWs, social workers or social work students, research assistants, nurses, and medical assistants—participated in 1 of 6 focus group sessions. Participants were aged between 21 and 70 years. Most identified as female (15/18, 83%), were not Hispanic/Latino (11/18, 61%), and had bachelor’s degrees (8/18, 44%). Table 1 summarizes the demographic characteristics of the participants.

**Table 1:** Participant characteristics (N=18).

Characteristics		Participants, n (%)
**Gender**		
	Male	2 (11)
	Female	15 (83)
	Nonbinary	1 (6)
**Age (y)**		
	20-29	8 (44)
	30-39	3 (17)
	40-49	3 (17)
	50-59	2 (11)
	60-69	1 (6)
	70-79	1 (6)
**Race**		
	Asian	2 (11)
	Black/African American	7 (39)
	Native Hawaiian or Pacific Islander	1 (6)
	White	7 (39)
	Prefer not to say	1 (6)
**Ethnicity**		
	Hispanic/Latino	7 (39)
	Not Hispanic/Latino	11 (61)
**Educational level**		
	High school graduate or equivalent	2 (11)
	Some college or certificate program	7 (39)
	Bachelor’s degree	8 (44)
	Doctorate degree	1 (6)
**Role**		
	Clinical manager	1 (6)
	Social worker	1 (6)
	Nurse	3 (17)
	Social work student	4 (22)
	Research assistant	2 (11)
	Medical assistant	3 (17)
	Community health worker	4 (22)

Most participants (11/18, 61%) did not have mental health training before the EMPOWER training but interacted with individuals with mental illness regularly during their work (Table 2).** **None of the participants had experience delivering structured BA therapy.

**Table 2:** Mental health (MH) training (N=18).

		Participants, n (%)
**Previous MH training**		
	Yes	7 (39)
	No	11 (61)
**Years of MH training**		
	0	13 (72)
	1	3 (17)
	2	2 (11)
**Experience delivering structured psychosocial therapies**		
	Yes	0 (0)
	No	18 (100)
**Average number of clients with MH issues interacted with per month**		
	0	7 (39)
	1-9	6 (33)
	10-19	2 (11)
	20-29	2 (11)
	30-39	0 (0)
	40-49	1 (6)

Table 3 summarizes the participants’ current technology use. Less than half (7/18, 39%) of the participants owned Android-supported devices. Participants were provided with Android tablets if they reported not having or being able to use an Android device during the focus group session; 28% (5/18) of the participants installed the app on their own devices, and the remaining participants were provided with Android tablets for this study.

**Table 3:** Technology use (N=18).

			Participants, n (%)
**Number of hours spent on a computing device (daily)**			
	0-3	0 (0)	
	4-6	4 (22)	
	7-9	9 (50)	
	10-12	3 (17)	
	≥13	2 (11)	
**Frequency of internet use**			
	At least once a day	2 (11)	
	Multiple times a day	5 (28)	
	Several times a day	1 (6)	
	Most of the day	10 (56)	
**Confidence using electronic devices**			
	Somewhat confident	4 (22)	
	Very confident	14 (78)	
**Owns an Android device**			
	Yes	7 (39)	
	No	11 (61)	

Overall, participants found the app usable, with an average SUS of 72.5 (SD 18.1) compared with the industry benchmark of 68, which has been found to be the average SUS for digital health apps and used in previous digital health studies [56,57]. The average SUS scores from older participants (aged >50 years) were lower than those from younger participants (Table 4). The NPS was 0 (neutral), with an equal distribution of promoters, detractors, and passives (6/18, 33% per NPS category).

**Table 4:** System Usability Scale score (N=18).

		Participants, n (%)	Scores, mean (SD)	Scores, median (IQR)
Total		18 (100)	72.5 (18.1)	71.3 (57.50-90)
**Age (years)**				
	20-29	8 (44)	74.7 (18.4)	72.5 (60-91.25)
	30-39	3 (17)	79.1 (20.2)	82.5 (57.5-97.5)
	40-49	3 (17)	73.3 (11.3)	72.5 (62.5-85)
	≥50	4 (22)	62.5 (22.7)	57.5 (45-80)
**Educational level**				
	High school or some college	9 (50)	74.4 (20.7)	82.5 (57.5-92.5)
	Bachelor’s degree or higher	9 (50)	70.5 (16.0)	70.0 (62.5-75.0)

### Perceived Feasibility of the App

In this section, we discuss the perceived feasibility of the app, which includes structural factors such as the current administrative and operational capabilities of health systems that could influence the introduction of the app. The participants discussed their current client-provider engagements and supervision mechanisms that could affect the potential introduction and incorporation of the app into current workflows and across the existing health system.

#### Client-Provider Engagement

Participants described how they worked with clients, which revealed potential considerations of how the app may need to be flexibly designed to support the ways in which FLWs meet with clients today. First, although in-person visits were still common, many participants shared that they engaged with their clients via telehealth. Furthermore, when meeting remotely with clients, participants shared that they may not necessarily have a quiet, private space to speak with clients and connectivity may be less reliable. Thus, obtaining high-fidelity recordings of sessions conducted in settings with frequent disruptions is an important consideration. Second, FLWs have limited time to interact with clients, and some participants shared that additional time would be needed to deliver a brief psychosocial intervention. Third, in some cases, screenings such as the Patient Health Questionnaire–9 for depression are currently administered and updated in electronic health record (EHR) systems such as Epic, and it may not be the FLW’s role to access, collect, or update these data even though such screening data may be needed for the delivery of a psychosocial intervention such as BA. When discussing client engagement, one participant noted the following:

...the social worker is the first person that meets with the patient because she talks to the patient to see what barriers they have to their personal care, which would involve depression screening, and any kind of behavioral health barriers that they may have going on. So that’s how we get our information about what the patient is kind of going through and what they need as far as behavioral health management.

P04; nurse

The integration of the app into existing EHR systems was reported as another important consideration.

Finally, although we did not specifically collect feedback on supervisors’ perceptions of the app, participants in supervisory roles suggested that additional time would be needed not only to review all the recordings but also to actually deliver the intervention—time that FLWs may not have:

From my role, like I said before, I don’t know that I would have the time to incorporate something like this into a patient encounter just because, like I said, we’ve got about 15, 20 minutes to see a patient for whatever issue is happening. And generally, when I see a patient for depression, anxiety, or something mental health, we certainly talk about it, talk about the role of treatment, maybe some options for counseling, not necessarily just medication. But I feel like when they get to me, it’s more about treatment and not so much about talking through things just because they’re not going to have as much time with a physician or a nurse practitioner because of their schedules.

P08; nurse practitioner

#### Supervision Structure

According to the participants, the current supervision structure includes structured supervisor-supervisee meetings scheduled as a part of annual performance reviews. In most settings, although they are not perceived as part of supervision and are not standardized, peer support groups are also available. Although the existing peer support mechanisms are informal and do not follow a structure similar to the supervisor-supervisee meetings, they function as a good support mechanism for team members in which “a doctor can support a doctor, a manager can support a manager, or even their employees” (P06; clinical manager). A participant noted the following:

We do huddles where we kind of discuss different things that are going on and changes within our settings and our clinical practice that are similar, but we don’t necessarily do any ratings.

P16; certified medical assistant

Thus, introducing the app would mean integrating these traditionally siloed supervisor-supervisee and peer support mechanisms and potentially introducing a new workflow that allows additional time and resources to be invested in supporting FLWs.

### Acceptability of the App

On the basis of feedback from the focus group sessions, participants’ overall impressions of the app were mainly positive. Participants reported that they found the app “easy,” “useful,” and overall “important” to their current workflows. The app could provide a much needed structured platform to support peer supervision and overall training of FLWs. A participant with no training in mental health care delivery before the BA training program noted the following:

I don’t have to be a licensed professional counselor, and I can actually use it myself to help and...get that supervision from my other peers to help me along the way.

P07; certified medical assistant

Another participant described the app and process as an “objective way to actually hear the session and hear how much each person reacted, how they delivered the information to the person, how the patient received it” (P08; nurse practitioner). She added that, as a supervisor, she could instruct the trainee to “go ahead and go do it, and then we’ll listen, and we can talk about feedback together.”

Even though most participants did not necessarily review or remember information from the app-related training materials provided to them before the focus group sessions, they considered the app easy to use. Participants who accessed the app on their devices found it easy to install. Most respondents also found the app navigation to be easy and self-explanatory. Nonetheless, it was noted that it could be a bit confusing at first “because you don’t know where to click,” (P02; social worker or social work student) and there were a few features within the app that the participants found more challenging to navigate than others (eg, navigating and interpreting different graphs and reviewing self-ratings). Participants needed time to familiarize themselves with the app and use it comfortably as they had never used a similar app before. One FLW reported the following:

I watched the (training) videos a long time ago and didn’t remember exactly everything that was talked about in the videos. And so, there’s a little bit of difficulty at first, but it’s pretty intuitive [as to] where everything is and how to use it. Or at least for me, it was, and so I could be able to grasp onto it pretty easily.

P03; social worker or social work student

The features that participants needed the most time to navigate included accessing recordings, graphs, and self-ratings.

### Perceived Usefulness of the App

Participants generally perceived the app to be potentially useful for a few reasons. One respondent shared that such an app would be useful to have now to address gaps in care because of limited access to and availability of mental health specialists:

For me, it would be a great thing to refer someone to if this was—if this was available because I think right now our psychiatrists are scheduling out next year. I think it’s a year before you can get the next appointment. So obviously, mental health is a big problem.

P08; nurse practitioner

The app could also help enhance FLWs’ job performance, highlight their areas for growth, and ensure overall intervention fidelity. The app’s ability to record and allow users to review and rate sessions can allow for more tailored feedback on performance enhancement, highlighting areas of growth:

I think as a supervisor, being able to record sessions could be really impactful if you have group supervision that you’re doing. Because if you have the consent from the patient and you’re able to kind of play this recording for everybody, you could kind of talk about what things went good [sic], areas of growth, basically. And so being able to use a recording, I think, could be helpful, as well as peer ratings and getting the ratings from your supervisor to kind of also see what areas of growth that you might have for behavioral activation or any other skills that you’re using at your place of employment.

P03; social worker or social work student

In addition to individual performance enhancement, the app could ensure intervention fidelity. A CHW shared that counseling sessions were often interrupted in the hospital setting; therefore, audio recording or rating could verify that the sessions were comprehensive despite disruptions. Reviewing the recording asynchronously could also help FLWs ensure that their sessions adhere to the intervention protocol.

Finally, most participants mentioned how their current peer support groups lacked structure and did not use systematic guidance or frameworks such as ratings for peer supervision. Respondents highlighted the potential of this app to fill that gap; however, the challenge would be in how to systematically incorporate the use of the app across the health systems currently implementing or planning to implement task-shared mental health interventions. As aptly summarized by a CHW, “to say that we need something like this, we definitely do. It’s just ‘where-do-we put-it type’ of situation” (P17; CHW).

### Barriers to and Facilitators of Scalability

Following discussions on the app’s perceived feasibility, usefulness, and acceptability, we probed considerations that could help the app’s broader implementation, including its fit into the FLWs’ current workflows and recommendations for future implementation.

#### Addressing Privacy Concerns

Protecting client privacy was expressed as a top priority for most respondents. Although feedback was mixed about clients’ apprehension toward audio-recorded sessions, it was generally agreed that obtaining proper client consent before recording was critical and necessary. Participants shared that consent from both FLWs and clients would be essential to ensure transparency and that all parties were informed of and agreed to how recordings would be used. Suggestions for increasing client comfort and obtaining adequate consent included building a trusted relationship with the client before asking to record sessions. By doing this, clients may be less hesitant to be recorded and more comfortable sharing their potential concerns.

Finally, regarding physical infrastructure, as noted previously, there is not always a private, physical space for FLWs to engage with clients and conduct peer supervision activities. One participant shared the following:

...we would probably talk about a lot of stuff that people would really want to be kept private with no real chance of anyone else hearing it, but that space is a big issue. We have a lot of shared offices—a lot of people in the same room at the same time...it might be better for us to, say, record a session so that we can make sure we’re getting everything, make sure I’m not missing something because that person’s over here, this person’s doing this, this person interrupted me, that person bumped into me. Yeah, we would just need to find a way to do it where we could minimize distractions, interruptions, and that kind of stuff.

P17; CHW

Another participant agreed, stating the following:

I’m in a room that can accommodate 25 people. And I have people all around me, and we’re all talking about different things. And it’s really hard.

P18; CHW

#### Ensuring the App Fits Workflow and Time Capacity

Although the app was perceived as an important addition to support their work, some participants raised concerns about finding time to deliver brief interventions and reviewing and discussing self- and peer performances. It was suggested that the ability to review and rate recordings anytime and anywhere is an advantageous feature of an app accessible from any device and that FLWs may prefer different device types depending on their environment or workflow. For example, one FLW noted the following:

I think mostly it would be done at a computer because we have very set clinic hours where I’m at. But in some other environments, I could see somebody trying to access it from a car or something like that.

P16; certified medical assistant

Another commented that, for her role, “...these (tasks) would be more flexible in very small devices, like iPad or phone, rather than recording it and everything, doing it in a computer” (P10; nurse). Asynchronous review would also allow for more flexibility in completing these follow-up activities:

I think being able to kind of reference the sessions that you’ve recorded will kind of allow for being a little more flexible in the schedule. Right now, you just have to go with each patient as it is, but this will allow...you to refer back. So, you don’t always have to just do it all at one time.

P16; certified medical assistant

Collecting and managing data offline would also be critical for FLWs working in areas with limited connectivity.

#### Providing Structured, Supportive Supervision

Respondents noted how the app could help supervisors give structured feedback to their staff, identify and highlight growth areas, and delegate appropriate tasks to FLWs based on their performance and expertise. Similarly, peer supervision could allow for more structured feedback from peers in workplaces that currently have informal peer support groups. Respondents confirmed that structured supervisor feedback could help with performance enhancement and quality assurance, particularly during the training phase, when workers need the most support. One participant noted the following:

...it would be good to be able to listen to how we interact with the patients. And it would be good to listen to it with the provider so...the nursing staff would know what we could do different, if we did well, just to get an idea of how we’re doing and how the patients are reacting to what we do.

P09; medical assistant

Participants felt that current app-supported supervision could allow for a more consistent feedback mechanism, systematic tracking of gradual performance improvement, and a constant feedback loop to enhance learning opportunities for FLWs.

Similarly, although respondents reported that informal peer feedback is available in some settings, it is largely unstructured. Most participants agreed that having their peers listen to their sessions and provide feedback is beneficial. Respondents were generally receptive to their peers being involved in supervisory processes, primarily as the participants viewed peer supervision more as “feedback” rather than a performance appraisal. However, it was suggested that it would be important, if not critical, to ensure that peers provide constructive and positive feedback to each other; concerns were raised about how the care team culture may be affected by peers giving and receiving negative ratings and critiques on performance. When asked how they would feel about their peers listening and rating their sessions, one participant noted that it is “beneficial to have another set of ears as long as it’s constructive criticism or constructive help” (P18; CHW). Regarding constructive criticism, “I think it’s all in bettering us so that we can better our patients, so that’s good” (P16; certified medical assistant). For some, ratings were only associated with yearly performance reviews with a supervisor. Respondents emphasized the importance of a nurturing and supportive peer group rather than a competitive one. Finally, participants agreed that the app had the potential to help with more structured self-assessments as well. The ability to record and review sessions could allow for more flexible self-evaluation and help with self-improvement and retrospective performance assessment.

## Discussion

### Principal Findings

We conducted this study to assess the feasibility and acceptability of a prototype app developed for FLW supervision using objective, metric-based performance evaluation and supportive feedback mechanisms in the United States. Participants found the app easy to use and acceptable. It was considered useful for supporting task sharing of mental health care services, enhancing FLWs’ skills, ensuring intervention fidelity, and providing structure to the traditionally unstructured peer support groups by introducing peer ratings and structured feedback mechanisms. Some key feasibility issues revealed in discussions with FLWs and stakeholders included (1) how the ability to record sessions with high fidelity may depend largely on the nature of the client encounter, (2) the potential need to integrate the app into an existing EHR system (eg, Epic), and (3) the introduction and integration of a new app-supported peer supervision workflow into existing supervisor-supervisee processes and peer support mechanisms. Among areas of improvement for scalability were privacy considerations both on recording and rating the session and ensuring the availability of private space in the health facility to conduct the mental health intervention successfully. Other areas of improvement included focusing on the additional time commitment required of FLWs and supervisors when introducing the app, including time to record and rate the sessions. Findings from our study highlight the app’s potential to catalyze the existing traditional supervisor-supervisee dyad model by shifting focus to a supportive supervision model that incorporates feedback not just from supervisors but also from peers and through self-assessments [44].

We would like to highlight 2 key findings that affect the scalability of the current app and similar digital tools that aim to support the monitoring of psychosocial intervention delivery through objective, measurable metrics. The first is the utility of the current app for supportive supervision of FLWs in the United States. Given the current focus on the scalability of task-shared mental health interventions and the need to ensure the quality delivery of task-shared psychosocial interventions, digital solutions such as the one proposed in this study are crucial [46,58]. Worldwide, digital interventions have shown great promise in supporting FLWs [59], and studies are now exploring digital supportive supervision more extensively [60,61]. The current app can rapidly scale this process, standardize performance measures, and account for evolving training needs with great flexibility. In this study, we demonstrated that an app based on work with nonspecialist providers in Madhya Pradesh and Goa, India, was found to be usable and potentially useful for FLWs in Texas and shows promise for supporting the scale-up of services delivered by FLWs in similar settings across the United States.

Another key finding of this study is the potential of the app to help merge traditionally siloed supervisor-supervisee and ad hoc peer feedback approaches to build a more unified, structured supervision process across existing health systems. This requires effort beyond adding the app to the existing workflow; rather, it may be necessary to develop a new workflow entirely. Participants expressed concerns about the additional time commitment required to use the app as a part of their job. However, it is important to note that the time constraints mentioned were not necessarily related to the use of the app itself but to adding objective, measurable metrics to assess FLWs’ performance. Introducing measurable metrics to evaluate performance requires recording, rating, and discussing sessions; additional time; and skill sets often beyond the FLWs’ job description. The time commitment required can constrain effective supervision in task-shared projects [29]. Therefore, the app’s scalability depends greatly on creating a workflow that benefits FLWs and communicating its need to support FLWs and task-sharing mental health care across health systems. Creating protocols that outline how to record sessions safely and securely for quality assurance is also critical, particularly in light of the privacy concerns associated with recording internet-based sessions [62]. Ensuring readiness for a systematized supportive supervision process guided by infrastructure and protocols contextualized for a particular type of setting is crucial for the success of task-shared mental health care delivery [63]. More importantly, the current app must flexibly align rather than conflict with existing supervision models and diverse FLW workflows to ensure that it truly supports FLW skill enhancement, effective feedback mechanisms, and adequate fidelity in task-shared interventions [64-66].

### Recommendations for Future Implementation

On the basis of the feedback collected in this study, there are several key considerations, challenges, and proposed solutions for the future implementation of this app. Table 5 summarizes the key considerations raised during the preliminary prototype design of the app, including advisory panel discussions, usability testing, and focus group session interviews. We propose solutions for each consideration that can inform future studies aimed at introducing similar digital tools to assist FLWs. These recommendations will also facilitate organizations’ development of supportive and structured supervision mechanisms.

**Table 5:** Key considerations, challenges, and proposed solutions for improvement.

Key considerations		Challenges	Proposed solutions
**System level**			
	Target users	App must support a variety of FLW^a^ roles with different workflows and role requirements as well as their supervisors	Design the app to include role-specific features that can be customized to support site-specific needs as allowed (eg, inclusion of depression screening)
	Integration into the current systems	Potential to increase workload associated with integrating measurement-based peer supervision into current workflows	Integrate the new app with existing EHRs^b^ such as Epic, which can reduce data redundancy and support more comprehensive client care
	Interoperability across operating systems	Prototype is currently only supported as a web application or Android mobile app	Ensure that the app is easily accessible and can support offline data collection on other commonly used devices (eg, iPhones)
**Organization level**			
	Available resources in the implementing health facilities	Inadequate physical space for conducting private and secure counseling sessions Lack of current protocols on supportive supervision to guide FLWs Need for structured peer supervision to implement the app	Assess the availability and accessibility of facilities’ resources before implementing the app Facilitate the development of protocols or updates to current protocols to promote the use of the app Set up or use the existing structured peer supervision mechanisms to support app integration
	Peer supervision model	More structured peer supervision could be cumbersome and time-consuming, especially for FLWs who are used to feedback from supervisors and are more comfortable with informal peer feedback mechanisms	Include specific training and guidance on delivering and receiving peer feedback Ensure supervisor buy-in and system support
	Hybrid workflow	Both in-person and remote (telehealth) client visits are common	Ensure the app can flexibly support recordings either directly in-app or via integration with other tools that may already be used
**App related**			
	Peer ratings	Introducing a mechanism for peers to rate and critique each other may feel uncomfortable	Include positive and encouraging language and features (eg, badges) to support improvement in performance Include space for qualitative feedback to provide more context for ratings
	Navigation	Navigation through parts of the app remains cumbersome	Enhance the app to support the following: Unnested features (eg, client or patient lists) An easy way to view completed self-ratings A way to flag incomplete self- and peer ratings Locating a session recording via multiple search parameters, such as client or patient ID, peer, or date
	Data visualization	Graphs and self-ratings are not intuitive	Simplify current features such that graphs are more easily accessed and usable across device screen sizes Highlight and indicate change in performance over time, noting specific areas that may need improvement

^a^FLW: frontline health worker.

^b^EHR: electronic health record.

### Conclusions

In summary, we demonstrated the feasibility and acceptability of a usable and functional mobile app prototype to support FLW-delivered psychosocial interventions in the United States. However, the success of future implementation hinges on further development of the app to ensure its adoption by both FLWs and their supervisors and overall readiness to adopt a novel peer supervision model of care. Feedback from stakeholders and target end users (FLWs) suggests that the app can help structure supervision mechanisms and systematize the collection and review of performance metrics in task-shared mental health interventions. With appropriate organization- and system-level support coupled with strong FLW training programs, the proposed app has the potential to scale up the delivery of high-quality, evidence-based mental health interventions in the United States through the critical role of FLWs who are already connected with and best positioned to serve some of the hardest-to-reach communities with the greatest needs for psychosocial support and care.

## Appendix

Multimedia Appendix 1 Focus group discussion task list.

Multimedia Appendix 2 COREQ (Consolidated Criteria for Reporting Qualitative Research) checklist.

## Abbreviations

BA: behavioral activation
CHW: community health worker
COREQ: Consolidated Criteria for Reporting Qualitative Research
EHR: electronic health record
FLW: frontline health worker
NPS: net promoter score
PEERS: Promoting Effective Mental Healthcare Through Peer Supervision
SUS: System Usability Scale

## Data Availability

The data sets generated during and analyzed during this study are available from the corresponding author on reasonable request.
