# Supplementary material for: Designing an App to Support Measurement-Based Peer Supervision of Frontline Health Workers Delivering Brief Psychosocial Interventions in Texas: Multimethod Study
Source: JMIR Form Res. 2024 Mar 11;8:e55205. doi: 10.2196/55205 (PMC10964140; doi:10.2196/55205)
Supplement: Multimedia Appendix 1 [file formative_v8i1e55205_app1.docx]

**Supplementary File: Focus Group Discussion Task List**

*Scenario: You have been trained in measurement-based peer supervision and you are being asked to use an app for your work.*

**Task 1: Using your computer, go to the web app (preferred browsers: Chrome, Firefox) using the link shared with you.**

**Task 2: Sign into the web app with your username (e.g., peer12) and password (e.g., 123).**

*Scenario: You have a new client who needs to be registered.*

**Task 3: Register the client named “Maria Lopez.”**

*Scenario: You have completed a session with a client and rated it. Now you want to see how you rated your own session.*

**Task 4: View your ratings for Treatment Specific Skills and General Skills for one of your recorded counseling sessions that you have already rated.**

**Task 5: For this same session, view the summary of your ratings in Session Rating Graphs.**

*Scenario: Your supervisor has assigned you a peer’s session to review and rate. This session recording is 12-15 minutes*.

**Task 6: Listen to the first 5 minutes of your peer’s recorded session.**

**Task 7: Complete and submit your ratings for the session.**

*Scenario: Now, you want to see how your supervisor and peers rated your sessions.*

**Task 8: For Jane Doe, view the average ratings submitted by yourself, your peers, and supervisor. Report the average ratings for the first session displayed on the Treatment Skill History bar chart.**

**Task 9: View the time-series graph with your overall performance as rated by your supervisor. Report the highest score for both Treatment Skills and General Skills.**

*Scenario: It is time to conduct a counseling session with your client, Maria Lopez. She has agreed to being recorded.*

**Task 10: Log into the Peer Supervision app on your Android device.**

**Task 11a: Pick a client and conduct a new session with them.**

**Task 11b: Record and save the phrase: “This is a test session for the Peer Supervision study.” Do not submit.**

**Task 11c: Play back the recording to make sure the recording was captured.**

**Task 11d: Enter comments about any potential issues that you may have encountered during your recording session with your client.**
