# Supplementary material for: Designing an App to Support Measurement-Based Peer Supervision of Frontline Health Workers Delivering Brief Psychosocial Interventions in Texas: Multimethod Study
Source: JMIR Form Res. 2024 Mar 11;8:e55205. doi: 10.2196/55205 (PMC10964140; doi:10.2196/55205)
Supplement: Multimedia Appendix 2 [file formative_v8i1e55205_app2.docx]

**Supplementary file: Consolidated criteria for reporting qualitative studies (COREQ)**

Developed from: Tong A, Sainsbury P, Craig J. Consolidated criteria for reporting qualitative research (COREQ): a 32-item checklist for interviews and focus groups. International Journal for Quality in Health Care. 2007. Volume 19, Number 6: pp. 349 – 357

| **No. Item** | **Guide questions/description** |  |
| --- | --- | --- |
| **Domain 1: Research team and reflexivity** |  |  |
| **Personal Characteristics** |  |  |
| 1. Inter viewer/facilitator | Which author/s conducted the interview or focus group? | YXH, DML |
| 2. Credentials | What were the researcher’s credentials? E.g. PhD, MD | PhD, BA |
| 3. Occupation | What was their occupation at the time of the study? | Director, Research Assistant |
| 4. Gender | Was the researcher male or female? | Females |
| 5. Experience and training | What experience or training did the researcher have? | YXH has a PhD degree and is trained in mixed methods, with extensive experience in qualitative research and peer-reviewed publications. DML has been trained in qualitative research and has experience leading one-on-one in-depth interviews and qualitative analyses. |
| **Relationship with participants** |  |  |
| 6. Relationship established | Was a relationship established prior to study commencement? | None of the participants had an established relationship with any of the authors prior to study commencement. |
| 7. Participant knowledge of the interviewer | What did the participants know about the researcher? e.g. personal goals, reasons for doing the research | Participants were informed that the researchers were interested in determining the usability and acceptability of the app. |
| 8. Interviewer characteristics | What characteristics were reported about the inter viewer/facilitator? e.g. Bias, assumptions, reasons and interests in the research topic | A brief introduction about the study, organization and interviewer’s names and positions provided to the participants before starting the interview. |
| **Domain 2: Study design** |  |  |
| **Theoretical framework** |  |  |
| 9. Methodological orientation and Theory | What methodological orientation was stated to underpin the study? e.g. grounded theory, discourse analysis, ethnography, phenomenology, content analysis | Thematic analysis |
| **Participant selection** |  |  |
| 10. Sampling | How were participants selected? e.g. purposive, convenience, consecutive, snowball | Convenience sampling was used. |
| 11. Method of approach | How were participants approached? e.g. face-to-face, telephone, mail, email | Focus Group Discussions (FGDs) were conducted. |
| 12. Sample size | How many participants were in the study? | 18 |
| 13. Non-participation | How many people refused to participate or dropped out? Reasons? | None |
| **Setting** |  |  |
| 14. Setting of data collection | Where was the data collected? e.g. home, clinic, workplace | All interviews were conducted remotely via conferencing. |
| 15. Presence of non-participants | Was anyone else present besides the participants and researchers? | No |
| 16. Description of sample | What are the important characteristics of the sample? e.g. demographic data, date | Potential end users of the app (i.e., frontline health workers) |
| **Data collection** |  |  |
| 17. Interview guide | Were questions, prompts, guides provided by the authors? Was it pilot tested? | FGD guide was prepared and rigorously discussed among the authors and probes were created to facilitate discussions. |
| 18. Repeat interviews | Were repeat interviews carried out? If yes, how many? | No |
| 19. Audio/visual recording | Did the research use audio or visual recording to collect the data? | Audio recorded |
| 20. Field notes | Were ﬁeld notes made during and/or after the interview or focus group? | Notes were taken during the discussion and were included as memos during analysis. |
| 21. Duration | What was the duration of the inter views or focus group? | 1.5 hours |
| 22. Data saturation | Was data saturation discussed? | No |
| 23. Transcripts returned | Were transcripts returned to participants for comment and/or correction? | No |
| **Domain 3: analysis and ﬁndings** |  |  |
| **Data analysis** |  |  |
| 24. Number of data coders | How many data coders coded the data? | Two authors (YXH and DML) coded the data. |
| 25. Description of the coding tree | Did authors provide a description of the coding tree? | Three authors (YXH, DML, and AP) generated a coding system and codebook in with domains, codes, and definitions to guide the coding process. |
| 26. Derivation of themes | Were themes identiﬁed in advance or derived from the data? | Themes were derived from the data. |
| 27. Software | What software, if applicable, was used to manage the data? | Dedoose (v. 9.0.107) |
| 28. Participant checking | Did participants provide feedback on the ﬁndings? | No |
| **Reporting** |  |  |
| 29. Quotations presented | Were participant quotations presented to illustrate the themes/ﬁndings? Was each quotation identiﬁed? e.g. participant number | Yes, quotations were presented to illustrate the themes/findings, and each quotation was identified with participant role and age. |
| 30. Data and ﬁndings consistent | Was there consistency between the data presented and the ﬁndings? | Yes |
| 31. Clarity of major themes | Were major themes clearly presented in the ﬁndings? | Yes |
| 32. Clarity of minor themes | Is there a description of diverse cases or discussion of minor themes? | Yes |
